# Redox Post-translational Modifications of Protein Thiols in Brain Aging and Neurodegenerative Conditions—Focus on S-Nitrosation

**DOI:** 10.3389/fnagi.2020.00254

**Published:** 2020-09-03

**Authors:** Mattéa J. Finelli

**Affiliations:** School of Medicine, Biodiscovery Institute, University of Nottingham, Nottingham, United Kingdom

**Keywords:** redox, post-translational modifications, cysteine residues, aging, neurodegenerative diseases, brain, S-nitrosation

## Abstract

Reactive oxygen species and reactive nitrogen species (RONS) are by-products of aerobic metabolism. RONS trigger a signaling cascade that can be transduced through oxidation-reduction (redox)-based post-translational modifications (redox PTMs) of protein thiols. This redox signaling is essential for normal cellular physiology and coordinately regulates the function of redox-sensitive proteins. It plays a particularly important role in the brain, which is a major producer of RONS. Aberrant redox PTMs of protein thiols can impair protein function and are associated with several diseases. This mini review article aims to evaluate the role of redox PTMs of protein thiols, in particular S-nitrosation, in brain aging, and in neurodegenerative diseases. It also discusses the potential of using redox-based therapeutic approaches for neurodegenerative conditions.

## Introduction

Reactive oxygen species and reactive nitrogen species (RONS) are essential signaling molecules produced by the aerobic metabolism (Friedman, 2010a; Finkel, 2011; Sbodio et al., 2019). One essential route to transduce RONS signaling is through oxidation-reduction (redox)-based post-translational modifications (PTMs) of proteins (Stadtman, 1988; Stadtman and Levine, 2003; Finkel, 2011; Santos and Lindner, 2017).

The mammalian brain is metabolically very active and is a major producer of RONS (Colton and Gilbert, 2002; Friedman, 2010a,b). Therefore, RONS-dependent redox signaling is particularly important in the normal physiology of the brain (Colton and Gilbert, 2002; Beckhauser et al., 2016). Under pathological conditions, RONS can reach excessive levels, generating oxidative and nitrosative (O/N) stresses, which can damage DNA, lipid, and proteins and be detrimental to cell function (Jones, 2006; Ray et al., 2012). O/N stresses are active contributing factors to the pathophysiological mechanisms underpinning neurodegenerative conditions including Alzheimer’s disease (AD), Parkinson’s disease (PD) and amyotrophic lateral sclerosis (ALS; Friedman, 2010a,b; Kim et al., 2015; Liu et al., 2017; Sbodio et al., 2019).

In aerobic organisms, RONS are constantly produced by the mitochondrial oxidative phosphorylation (OXPHOS) system and by several enzymes [e.g., NADPH oxidases (NOXs) and nitric oxide synthases (NOSs); Ignarro, 1990; Raha and Robinson, 2000; Forman et al., 2002; Bian and Murad, 2003; Murphy, 2009; Lambeth and Neish, 2014; Nayernia et al., 2014; Di Meo et al., 2016; Ma et al., 2017; Moldogazieva et al., 2018; Barua et al., 2019; Sbodio et al., 2019; Gantner et al., 2020]. Some of the major RONS include hydrogen peroxide (H_2_O_2_), hydroxyl radical (•OH), superoxide anion (O_2_^•−^), peroxyl radical (ROO^•^), singlet oxygen (^1^O_2_), nitic oxide or nitrogen monoxide (^•^NO), nitrogen dioxide (^•^NO_2_), and peroxynitrite (ONOO^−^; Commoner et al., 1954; McCord, 2000; Forman et al., 2002; Pham-Huy et al., 2008; Di Meo et al., 2016; Sbodio et al., 2019). A tightly regulated machinery of small molecules [e.g., cysteine, ascorbate, glutathione (GSH)] and proteins [e.g., superoxide dismutases (SODs), catalases (CATs), glutathione peroxidases (GP_X_s), peroxiredoxins (PRDXs)] reduce RONS to control their levels in cells (Forman et al., 2002; Birben et al., 2012; Moldogazieva et al., 2018; Paul et al., 2018; Sbodio et al., 2019). Under normal physiological (eustress) conditions, RONS production and reduction are balanced; however, any imbalance can generate O/N stresses (Jones, 2006; Moldogazieva et al., 2018; Sies, 2019). Crosstalk between ROS and RNS, their specific sources and scavengers regulate the distribution of the various RONS present in cells (RONS composition), their relative abundance and absolute levels (Forman et al., 2002; Daiber, 2010; Dikalov, 2011; Schulz et al., 2014; Daiber et al., 2017). This complex network of RONS triggers a downstream cascade of events, termed redox signaling (Forman et al., 2002, 2014; Jones, 2006; Ray et al., 2012; Moldogazieva et al., 2018).

This mini review article focuses on the signaling triggered by RONS and transduced through redox PTMs of protein thiols and highlights key features of this signaling in normal cell physiology. It also assesses the role played by this redox signaling in brain aging and neurodegenerative conditions. Lastly, it aims to argue for greater considerations of the complexities of this essential signaling when designing new redox-based therapeutic strategies for neurodegenerative diseases.

## Redox Post-translational Modifications (PTMs) of Protein Thiols and Their Role as a Redox Switch for Protein Function

**Figure 1:**
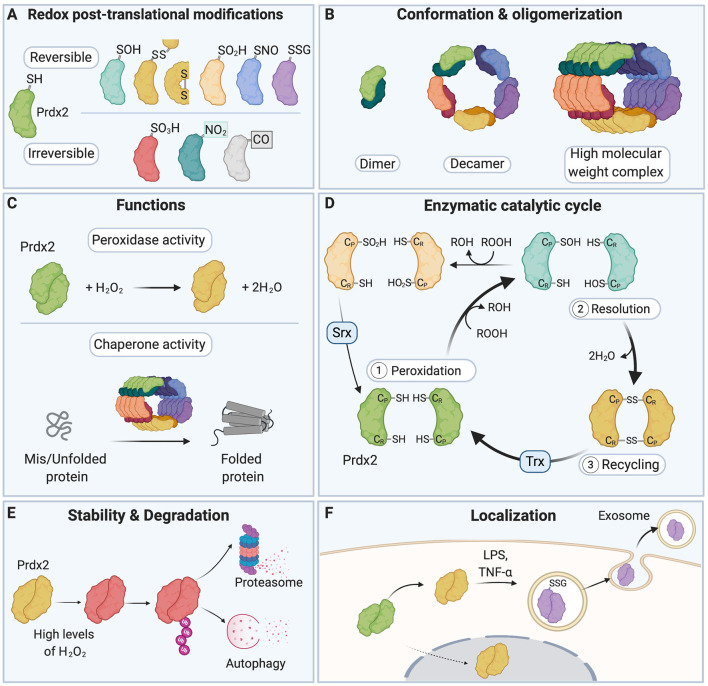
A redox switch modulates the multiple functions of Peroxiredoxin 2 (Prdx2). **(A)** The various residues of Prdx2 can be modified by S-sulfenylation (–SOH), intermolecular and intramolecular disulfide bonds (–SS–), S-sulfinylation (–SO_2_H), S-nitrosation (–SNO), S-glutathionylation (–SSG), S-sulfonylation (–SO_3_H), nitration (–NO_2_), and carbonylation (–CO: carbonyl groups). Only a subset of all the post-translational modifications (PTMs) that have been described for Prdx2 is shown here for clarity. **(B)** The redox state of the Cys residues of Prdx2 regulates its conformation as a dimer, decamer, or high molecular weight complex: the reduced and overoxidized dimers strongly tend to form decamers or high molecular weight complex, while oxidized forms are preferentially present as dimers. **(C)** Depending on its redox PTMs and conformation, Prdx2 can act as an antioxidant enzyme or as a chaperone. Multiple redox PTMs can coordinately regulate Prdx2 functions. **(D)** As part of Prdx2 catalytic cycle, its conserved peroxidatic cysteine (C_P_) first reduces H_2_O_2_ or ROOH (peroxidation step). Then a second free thiol (C_R_ or resolving cysteine) forms a disulfide bond with its C_P_ residue (resolution step). The catalytic cycle is completed when the disulfide bond is recycled, typically by a thioredoxin-like molecule (Trx), regenerating the free thiol forms of the C_P_ and the C_R_ residues (recycling step). C_P_-SOH Prdx2 can react with a second ROOH molecule before it can react with C_R_-SH, becoming overoxidized (C_P_-SO_2_H); this in turn inactivates Prdx2 peroxidase activity. C_P_-SO_2_H can be reduced by sulfiredoxin (Srx). **(E)** Oxidation of Prdx2 active site (Cys51) triggers conformational changes that bring to the protein surface of Prdx2 a flexible C-terminus that is subsequently polyubiquitinated; this leads to degradation of oxidized Prdx2 by the proteasome and autophagy. **(F)** Redox PTMs of Prdx2 can also regulate its secretion in the extracellular space. Upon exposure to inflammatory stimuli (e.g., LPS or TNF-α), Prdx2 Cys51 and Cys172 residues are oxidized forming disulfide-linked homodimers and mixed disulfides with glutathione, which triggers its secretion in exosomes into the extracellular milieu. Prdx2, peroxiredoxin 2; C_P_, peroxidatic Cys residue; C_R_, resolving Cys residue; LPS, lipopolysaccharide; TNF-α, tumor necrosis factor-α; Trx, Thioredoxin; Srx, Sulfiredoxin.

Proteins are the main targets of RONS in cells as a result of their high rate constants for oxidation reactions and their abundance (Stadtman and Levine, 2003; Davies, 2005, 2016; Wall et al., 2012; Moldogazieva et al., 2018). Redox PTMs can occur nearly on any amino acid side chains (Stadtman and Levine, 2003; Dickinson and Chang, 2011; Wall et al., 2012; Corcoran and Cotter, 2013; Go and Jones, 2013). Nitration can modify tyrosine residues, and carbonylation can modify lysine, arginine, proline and threonine residues; however, these modifications are generally stable or irreversible and associated with O/N stresses (Dickinson and Chang, 2011; Wall et al., 2012; Corcoran and Cotter, 2013; Houée-Lévin et al., 2015; Weng et al., 2017; Gonos et al., 2018). Redox PTMs of thiol-containing cysteine (Cys) residues are considered to be one of the main drivers of redox signaling in cells because these residues can be easily oxidized and their oxidation is mostly reversible, with rates of oxidation and reduction compatible with signaling initiation and transduction (Miseta and Csutora, 2000; Davies, 2005, 2016; Bindoli et al., 2008; Schöneich, 2011; Wall et al., 2012; Wani et al., 2014; Go et al., 2015). The electronic structure of the thiol group (–SH) of Cys residues enables multiple oxidation states from −2 to +6 leading to a range of reversible and irreversible redox PTMs (Reddie and Carroll, 2008; Wang et al., 2012b; Chung et al., 2013; Cremers and Jakob, 2013; Wani et al., 2014; Figure 1A). Suggesting a potential cross-regulation between different PTMs, Cys residues can be targeted by a number of redox PTMs; for instance, the active Cys sites of protein disulfide isomerase (PDI) can be S-nitrosated or S-glutathionylated (Halloran et al., 2013). Besides, an interconversion between redox PTMs can also occur, with sulfenic acid (–SOH) representing a potential precursor for other reversible redox PTMs, such as S-glutathionylation, or intramolecular disulfide bond formation, possibly through sequential thiol modifications (Gallogly and Mieyal, 2007; Mieyal et al., 2008; Poole and Nelson, 2008; Sabens Liedhegner et al., 2012). The precise mechanisms underlying cross-regulation and interconversion between different redox PTMs of protein thiols remain unclear and future studies investigating multiple redox PTMs in parallel and over appropriate time-course will be required to fully explore these important aspects of redox PTMs regulation.

Mass spectrometry-based redox proteomic studies that have mapped the redox-modified Cys residues across the proteome (redox Cys-proteome) have shown that, for a given condition, only a very specific subset of Cys residues is redox-modified, demonstrating that RONS modify Cys residues in a very selective manner (Doulias et al., 2010; Marino and Gladyshev, 2010, 2012; McDonagh et al., 2014; Gould et al., 2015; Raju et al., 2015; Araki et al., 2016; Sun et al., 2016; Topf et al., 2018; van der Reest et al., 2018; Mnatsakanyan et al., 2019; Xiao et al., 2020). This selectivity is modulated by several factors including the pKa of the –SH moiety of the Cys residue, its accessibility, the other PTMs present in the target protein, and the charges of the neighboring amino acids and local electrostatic effects (Hess et al., 2001; Davies, 2005; Doulias et al., 2010; Marino and Gladyshev, 2010, 2012; Chung et al., 2013; Roos et al., 2013; Gould et al., 2015; Sun et al., 2016; Mnatsakanyan et al., 2019; Xiao et al., 2020). Many potential consensus motifs have been identified and proposed as a chemical rationale that could underlie the redox sensitivity of specific Cys residues in a given protein (Stamler et al., 1997; Doulias et al., 2010; Marino and Gladyshev, 2010, 2012; Chen et al., 2014, 2015; Gould et al., 2015; Sun et al., 2016; Topf et al., 2018; Mnatsakanyan et al., 2019; Xiao et al., 2020). The variety of consensus motifs proposed so far may partly reflect the wide range of experimental and computational approaches employed to identify the protein thiols that are modified by redox PTMs. Indeed, several studies have focused on endogenous redox PTMs while others have studied redox PTMs induced by exogenously applied RONS (e.g., H_2_O_2_, S-Nitroso-l-cysteine, S-Nitrosoglutathione, etc.), and an array of proteomic approaches with varying degrees of sensitivity and selectivity has been used across studies. Thus, finding unifying motifs and rules overarching the specificity of redox PTMs on a proteome-wide scale remains an outstanding challenge. It has been proposed that under eustress, for a given RONS composition, RONS absolute levels, and relative abundance, only a subset of redox-sensitive Cys residues within a protein is potentially susceptible to several redox-PTMs. In contrast, O/N stresses can lead to aberrant redox PTMs including redox PTMs on even lowly-reactive Cys residues, and/or addition of excessive, persistent or irreversible redox PTMs (Chung et al., 2013). This continuum of reactivity of Cys residues in redox-sensitive proteins may provide a redox-sensing mechanism for cells to monitor RONS composition and RONS levels in the cellular environment and to coordinate an appropriate downstream response.

Redox PTMs on proteins act as a “redox switch” that dynamically regulates protein function (Go and Jones, 2013; Go et al., 2015; Fra et al., 2017). Redox PTMs can lead to structural changes in the target protein through disulfide bond formation, including intramolecular disulfide bonds, Cys-dependent metal cofactor interactions, and through alteration of the topography of the protein (Davies, 2005; Khoo and Norton, 2011; Wani et al., 2014). The way redox PTMs affect protein conformation is partly dictated by the position of the modified Cys residue in the target protein, e.g., on the protein surface or buried, or on the protein backbone or side-chains (Dean et al., 1997; Davies, 2005). It remains difficult to predict the precise effect of redox PTMs on protein structure and function, and thus computational and experimental approaches need to be combined to fully uncover the functional consequences of redox PTMs on a given protein (Raimondi et al., 2014; Wani et al., 2014). Redox PTMs can also modulate the stability and degradation of target proteins (Pajares et al., 2015, 2018). It has been suggested that depending on the degree of oxidation of a protein (esp. whether its redox PTMs are reversible or irreversible), degradation can occur through either ATP- and polyubiquitination-dependent or independent mechanisms (Pajares et al., 2015; Song et al., 2016). Redox PTMs can induce conformational changes that expose hydrophobic structures normally buried in the natively folded protein; these structures become recognition and binding surfaces for the 20S core subunit of the proteasome, which subsequently degrades the oxidized target protein (Grune et al., 1997; Jung and Grune, 2008; Jung et al., 2014). The redox state of protein thiols can also regulate the localization of target proteins (Wani et al., 2014). Importantly, redox PTMs can ultimately tune the activity of redox-sensitive enzymes and regulate their enzymatic cycle and recycling (Klomsiri et al., 2011). For instance, the function of the redox-sensitive protein, Prdx2, is regulated by a redox switch (Rhee and Kil, 2017). Depending on the cellular RONS composition and levels, the various residues of Prdx2 can be decorated by an array of different redox PTMs (Fang et al., 2007; Park et al., 2011; Engelman et al., 2013; Peskin et al., 2013, 2016; Wong et al., 2013; Randall et al., 2014, 2019; Salzano et al., 2014; Svistunova et al., 2019; Figure 1A). Redox PTMs of Prdx2 regulate its conformation (Wood et al., 2003; Barranco-Medina et al., 2009; Hall et al., 2009, 2011), catalytic cycle (Woo et al., 2005; Hall et al., 2009; Karplus, 2015; Rhee, 2016), degradation (Song et al., 2016) and secretion (Salzano et al., 2014; Mullen et al., 2015; Figures 1B–F). Crucially, redox PTMs can coordinately regulate Prdx2 multiple functions as an antioxidant enzyme and a chaperone. For example, overoxidation (Prdx2-SO_2/3_) or S-nitrosation (Prdx2-SNO) of Prdx2 can inactivate its antioxidant activity, while Prdx2-SO_2/3_ stabilizes Prdx2 high-molecular weight chaperone complexes (Wood et al., 2003; Jang et al., 2004; Fang et al., 2007; Hall et al., 2009, 2011; Rhee and Woo, 2011; Saccoccia et al., 2012; Rhee and Kil, 2017; Svistunova et al., 2019).

By acting as a redox switch for protein function, redox PTMs allow for proteins to carry out different functions in different subcellular compartments. Indeed, the nature of the redox PTMs on protein thiols is greatly determined by the RONS composition and pH of the environment surrounding the target protein (Pace and Weerapana, 2013; Roos et al., 2013). Therefore, when a protein shuttles to a different compartment with a different RONS composition and pH, the redox PTMs of the protein as well as its activity will change accordingly (Go and Jones, 2008; Jones and Go, 2010). Together, redox PTMs of protein thiols constitute a powerful mechanism that allows for a very dynamic and compartment-specific regulation of protein functions in cells.

## Role of Redox PTMs of Protein Thiols in Cell Physiology and Brain Aging

Advances in redox proteomics have allowed to explore further the functional role of redox PTMs of protein thiols in cell and organ physiology. In particular, a recent study has mapped the oxidized Cys residues landscape in ten different tissues from wild-type mice using a highly sensitive and specific approach (a Cys-reactive phosphate tag method) and has shown that oxidized Cys residues were detected in almost half of all proteins in all tissues tested suggesting that redox PTM of protein thiols is a widely used signaling mechanism in normal cellular physiology (Go et al., 2011; Xiao et al., 2020). Analyses of the redox Cys-proteome across subcellular compartments also demonstrated that redox PTM of protein thiols occurs in all subcellular compartments, from nucleus, cytoplasm, Golgi, endoplasmic reticulum (ER) to mitochondria (Go et al., 2011; Chung et al., 2013; Doulias et al., 2013; Xiao et al., 2020).

**Figure 2:**
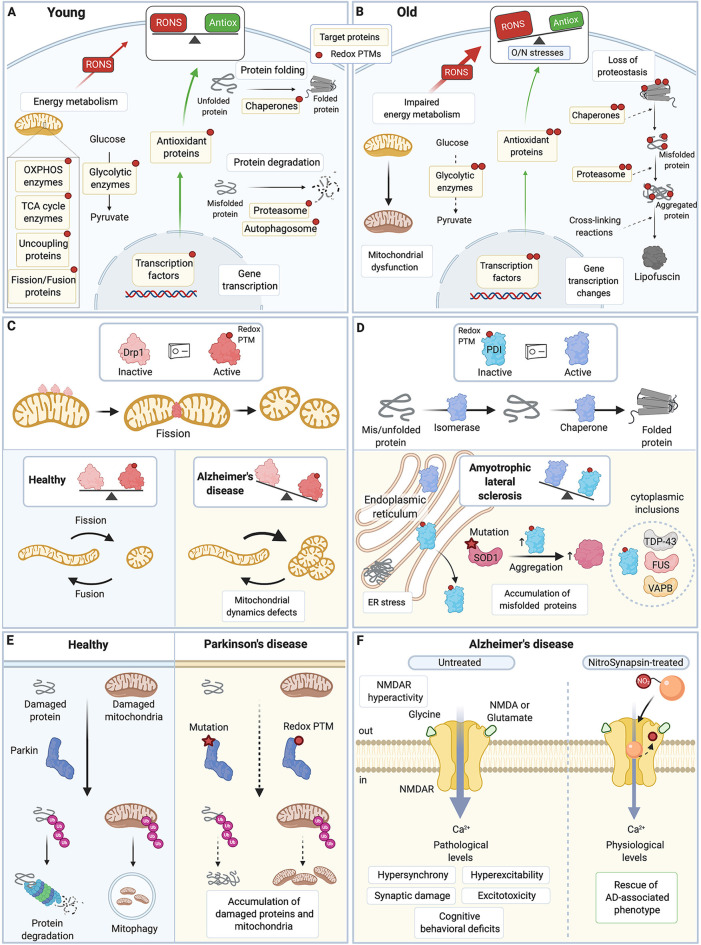
Redox PTMs of protein thiols are essential for normal cell physiology and contribute to aging and neurodegenerative diseases. **(A)** In young healthy cells, redox PTMs are an integral part of the normal cell signaling: they can coordinately modulate the activity of multiple proteins depending on the local RONS composition and levels. Redox PTMs regulate the function of proteins involved in essential pathways such as energy metabolism, protein folding and degradation, and gene transcription. Redox PTMs are represented by a red dot on the target proteins. **(B)** During aging, imbalance between RONS production and the antioxidant capacity of the cell leads to O/N stresses, and proteins may become differentially and/or aberrantly modified by redox PTMs, as represented by multiple red dots on the target proteins. These redox PTMs can affect the function of target proteins, ultimately altering key pathways that contribute to the aging process. **(C)** Drp1 is a GTPase protein involved in mitochondrial fission; when activated, especially by S-nitrosation, Drp1 forms oligomers that wrap around the mitochondrial outer membrane and scission it. Excessive levels of S-nitrosated Drp1, as seen in AD, disrupt the physiological equilibrium between mitochondrial fission and fusion,leading to increased mitochondrial fission, and synaptic and neuronal damage. **(D)** PDI is an ER protein with a dual isomerase and chaperone activity. S-nitrosation of PDI inhibits its functions. Excessive S-nitrosation of PDI, as observed in AD, PD, and ALS, leads to the accumulation of misfolded proteins in the ER and consequently, to increase in ER stress and neuronal cell death. In ALS, PDI is recruited to cytoplasmic inclusions containing ALS-associated proteins such as SOD1, TDP-43, FUS, or VABP. S-nitrosation of PDI also increases insoluble aggregates of ALS-associated mutant SOD1. **(E)** Parkin is an E3 ubiquitin ligase that targets damaged proteins and mitochondria to the ubiquitin-proteasome system and mitophagy. Loss-of-function mutations in Parkin are associated with familial PD. Importantly, excessive and persistent S-nitrosation of non-mutated Parkin, as detected in postmortem brains of patients with sporadic PD, also inhibits Parkin activity, which impairs ubiquitination of its substrate proteins and contributes to the accumulation of damaged proteins and mitochondria and ultimately to neuronal cell injury and death. **(F)** Hyperactivity of the extrasynaptic NMDAR leads to excessive intracellular calcium, hypersynchrony and hyperexcitability of neurons, and synaptic damage and excitotoxicity in AD. A new drug, NitroSynapsin, is constituted of a memantine moiety and an added nitro group (NO_2_). It can dampen the hyperactivity of NMDAR while preserving its physiological function by binding inside the excessively opened channel and by acting as an NO group donor, which triggers NMDAR inactivation through S-nitrosation of its redox-sensitive Cys residues. NitroSynapsin treatment significatively improves the pathological features associated with AD. TCA, tricarboxylic acid cycle; OXPHOS, oxidative phosphorylation; RONS, reactive oxygen species and reactive nitrogen species; Antiox, antioxidant capacity; Drp1, Dynamin related protein 1; PDI, protein disulfide isomerase; ER, endoplasmic reticulum; SOD1, superoxide dismutase 1; TDP-43, transactive response DNA binding protein 43 kDa; FUS, Fused in sarcoma; VAPB, vesicle-associated membrane protein; Ub, ubiquitin; NMDAR, N-methyl-D-aspartate receptor; AD, Alzheimer’s disease; PD, Parkinson’s disease; ALS, amyotrophic lateral sclerosis. All figures were created with BioRender.com.

Redox PTMs have been shown to regulate the activity of proteins implicated in a range of major cellular functions, and cell survival/death (Trachootham et al., 2008; Brigelius-Flohé and Flohé, 2011; Handy and Loscalzo, 2012; Wall et al., 2012; Mailloux et al., 2014; Niforou et al., 2014; Go et al., 2015; Jones and Sies, 2015; Pajares et al., 2015; Nakamura and Lipton, 2017; Figure 2A). In particular, redox PTMs play an essential role in the regulation of energy metabolism in cells through S-nitrosation, S-glutathionylation, or S-sulfenylation of proteins of the mitochondrial respiratory complexes I, II, IV, and V, and ATP synthase (Hurd et al., 2008; Garcia et al., 2010; Handy and Loscalzo, 2012; Wang et al., 2013; Mailloux et al., 2014; Nakamura and Lipton, 2017; van der Reest et al., 2018; Xiao et al., 2020), enzymes of the tricarboxylic acid cycle (e.g., Alpha-ketoglutarate dehydrogenase, Isocitrate dehydrogenase, Aconitase; Kil and Park, 2005; McLain et al., 2013; Yan et al., 2013; Bulteau et al., 2017; Nakamura and Lipton, 2017, 2020; Xiao et al., 2020), enzymes of glycolysis [e.g., Hexokinase, Glyceraldehyde 3-phosphate dehydrogenase (GAPDH); Riederer et al., 2009; Mailloux et al., 2014; McDonagh et al., 2014; Araki et al., 2016; van der Reest et al., 2018; Xiao et al., 2020] and of fatty acid metabolism (e.g., Very long chain acyl-coenzyme A dehydrogenase; Doulias et al., 2013). Similarly, for proteostasis, redox PTMs have been reported on chaperones (e.g., Heat shock protein 70, PDI; Wang C. et al., 2012; Grunwald et al., 2014), subunits of the proteasome (Aiken et al., 2011; Jung et al., 2014; Kors et al., 2019), and proteins involved in autophagy (e.g., Autophagy Related 3, 4 and 7; Frudd et al., 2018; Pajares et al., 2018; Scherz-Shouval et al., 2019), which ultimately contribute to the regulation of protein folding and degradation (Niforou et al., 2014; Pajares et al., 2015). Redox PTMs can also regulate the activity of several transcription factors (Brigelius-Flohé and Flohé, 2011). For instance, oxidation, S-nitrosation or nitration of p53, activating protein 1, myocyte enhancer factor 2 or nuclear factor kappa-light-chain-enhancer of activated B cells (NF-kB) modulate the DNA binding and activity of these important transcription factors (Abate et al., 1990; Schreck et al., 1991; Rainwater et al., 1995; Klatt et al., 1999; Kabe et al., 2005; Yakovlev et al., 2007; Jung et al., 2008; Ljubuncic et al., 2008; Okamoto et al., 2014; Caviedes et al., 2020). Redox PTMs can also affect the activity of transcription factors through indirect mechanisms. For example, redox PTMs on Kelch-like ECH-associated protein 1 (Keap1) trigger the dissociation of the cytoplasmic complex formed by Keap1 and nuclear factor erythroid 2-related factor 2 (Nrf2), which allows Nrf2 to translocate to the nucleus and to bind to the antioxidant response element of its target genes (Dinkova-Kostova et al., 2002, 2017; Rachakonda et al., 2008).

More globally, comprehensive functional analyses on the redox Cys-proteomes of various cell types and tissues have demonstrated that redox PTMs can regulate whole networks of proteins that share similar biological activities (Doulias et al., 2010; Go et al., 2011, 2015; Xiao et al., 2020). Importantly, this coordinated redox-based regulation of functional protein networks underpins, at least in part, the unique physiological and metabolic states that characterize the different cell types and tissues present in organisms. Indeed, redox PTMs of protein thiols can tune the activity of ubiquitously expressed proteins in a tissue-specific manner and can also regulate functional networks specifically in different tissues to control both tissue-ubiquitous functions (e.g., tRNA aminoacylation) and tissue-specific functions (e.g., catabolism of glycogen and glucose; Doulias et al., 2013; Xiao et al., 2020). Therefore, redox PTMs of protein thiols play an essential part in normal cell physiology and coordinately regulate functional networks in a tissue- and cell type-specific manner.

Aging is characterized by molecular and cellular hallmarks including, but not limited to, altered mitochondrial function, impaired energy metabolism, increased O/N stresses, and loss of proteostasis (López-Otín et al., 2013; Figure 2B). Many aspects of the fundamental molecular mechanisms of brain aging remain unknown (Mattson and Arumugam, 2018). The “free radical theory of aging” postulates that aging is driven by an accumulation of harmful and irreversible RONS-induced damage to DNA, lipids, and proteins (Harman, 1956, 2006). In accordance with this theory, an increase in modified proteins (e.g., S-nitrosated Cys residues, and nitrated and carbonylated amino acids) has been observed in the brain of aging rodents and humans, especially in the hippocampus, substantia nigra and frontal cortex, brain regions particularly vulnerable to aging-associated diseases (Smith et al., 1991; Cini and Moretti, 1995; Farr et al., 2003; Dremina et al., 2005; Siqueira et al., 2005; Poon et al., 2006b; Gokulrangan et al., 2007; Prokai et al., 2007; Riederer et al., 2009; Grimm et al., 2011; Venkateshappa et al., 2012; Ortuño-Sahagún et al., 2014; Perluigi et al., 2014; Cabré et al., 2017; Gonos et al., 2018). In addition, in line with an age-dependent increase in O/N stresses, accumulation of redox PTMs (e.g., S-sulfonylation and excessive disulfide bonds) on key antioxidant proteins such as PRDXs and SODs has been described and shown to impair the enzymatic activity of these proteins (Kurokawa et al., 2001; Navarro and Boveris, 2004; Poon et al., 2006a,b; Musicco et al., 2009; Pérez et al., 2010; Gottfredsen et al., 2013; McDonagh et al., 2014; Perluigi et al., 2014).

Irreversible redox PTMs can participate in the aging process by triggering unfolding and aggregation of proteins through hydrophobic and electrostatic interactions between target proteins (Höhn et al., 2017). With time, these aggregates can be covalently stabilized, rendering protein aggregates insoluble and non-degradable (Pacifici et al., 1993; Giulivi et al., 1994; Johnston et al., 1998; Calderwood et al., 2009; Koga et al., 2011; Höhn et al., 2017). Existing protein aggregates can undergo further cross-linking reactions with molecules such as aldehydes and malondialdehyde, products of lipid peroxidation, resulting in the formation of highly cross-linked autofluorescent material referred to as lipofuscin, an important hallmark of aging (Stadtman and Levine, 2003; Terman and Brunk, 2004; Jung et al., 2007). O/N stresses also impair the activity of the proteasome through irreversible redox PTMs such as carbonylation of its subunits, leading to an age-dependent reduction of protein degradation and further accumulation of protein aggregates (Friguet, 2006; Jung et al., 2014; Höhn and Grune, 2014; Lefaki et al., 2017). These protein aggregates may in turn interfere with cell homeostasis.

Also, aberrant redox PTMs of proteins on Cys and other residues may be implicated in the impaired energy metabolism observed in aging (Yap et al., 2009; Barzilai et al., 2012; López-Otín et al., 2013; Chistiakov et al., 2014; Jové et al., 2014; Bouzier-Sore and Bolaños, 2015; Goyal et al., 2017; Mattson and Arumugam, 2018; Rizza et al., 2018; Hipkiss, 2019; Ravera et al., 2019). For instance, 3-nitrotyrosine and carbonylation have been detected on energy-related enzymes, proteins of the mitochondrial respiratory complex and ATP-synthase in aged rats, in the senescence-accelerated (SAMP8) mouse model of aging, and in human postmortem tissue from aged control patients (Smith et al., 1991; Navarro and Boveris, 2004; Poon et al., 2004, 2006a,b; Gokulrangan et al., 2007; Lam et al., 2009; Perluigi et al., 2010; Perluigi et al., 2014; Pérez et al., 2010). Aberrant redox PTMs of proteins may also contribute to the age-associated reduced synaptic transmission and impaired induction of long-term potentiation (Foster and Norris, 1997; Foster and Kumar, 2002; Foster, 2006, 2007, 2012; Kumar et al., 2009, 2018; Bodhinathan et al., 2010a,b; Oh et al., 2010; Bradley and Steinert, 2016; Hidalgo and Arias-Cavieres, 2016; Muñoz et al., 2020). For example, the redox-sensitive ryanodine receptor 3 (RyR3) essential for synaptic transmission and plasticity, is nitrated in the cerebellum of aged rats (Gokulrangan et al., 2007; Lanner et al., 2010; Abu-Omar et al., 2018; Arias-Cavieres et al., 2018; Muñoz et al., 2020). Interestingly, hyper-S-nitrosation of RyRs shifts these receptors from a finely regulated state to unregulated Ca^2+^ leak channels (Bull et al., 2008; Bellinger et al., 2009; Bodhinathan et al., 2010b; Liu et al., 2012; Lacampagne et al., 2017; Muñoz et al., 2020). S-glutathionylation of RyRs also compromises their function (Bull et al., 2003; Hidalgo, 2005; Aracena-Parks et al., 2006). Together, this suggests that redox PTMs of RyRs may contribute to the progressive age-dependent dysregulation of synaptic transmission.

There is a growing body of evidence however that conflicts with the idea that RONS and associated redox PTMs of proteins in aging are solely detrimental to cell function. The “redox theory of aging” indeed presents the redox proteome as a system that allows organisms to best adapt to a changing environment and varied exposures (exposome) throughout their lifespan, and which contributes to building progressive resilience (Go and Jones, 2013; Jones, 2015; Jones and Sies, 2015). It also states that the process of aging could be a gradual breakdown of the redox proteome and redox metabolome, which act together as adaptive interfaces supporting the interaction between the genome and the exposome (Jones, 2015; Go and Jones, 2017). Other studies have also demonstrated that mitochondrial RONS can initiate a positive adaptive homeostasis cascade following a non-linear response (termed mitohormesis); both the dose and timing of RONS exposure determining whether RONS have either a beneficial or detrimental effect on cell function (Kawagishi and Finkel, 2014; Ristow, 2014; Ristow and Schmeisser, 2014; Yun and Finkel, 2014). Mild O/N stresses can indeed trigger a protective response that makes organisms more resistant to future, greater, oxidative insults and therefore increasing levels of RONS (and potentially of redox PTMs of protein thiols) can be beneficial at the cellular or organismal levels and can even promote healthspan and lifespan (Kawagishi and Finkel, 2014; Ristow, 2014; Ristow and Schmeisser, 2014; Yun and Finkel, 2014). It has been hypothesized that aging results in a generalized decline in this adaptive homeostasis, in particular through an age-dependent attenuation of proteolytic activity and capacity (Pomatto and Davies, 2018).

Besides, also contrary to the concept that RONS induce a bulk accumulation of non-specific redox PTMs of protein thiols in aging, a recent study that compared the redox Cys-proteome in tissues of young (16-week-old) and old (80-week-old) mice has shown that the overall levels of reversibly oxidized Cys residues are very similar in young and aged tissues (Xiao et al., 2020), which was consistent with previous observations made in *D. melanogaster* (Menger et al., 2015). Interestingly, it also found that young and aged tissues have largely distinct redox proteomic signatures with different highly modified Cys residues (Xiao et al., 2020). This demonstrates that a profound and very specific remodeling of the redox signaling landscape occurs during aging, which in turn induces tissue-specific functional shifts. It remains to explore however whether the redox PTMs that have been detected in aged tissue so far contribute to either accelerating or slowing down the the aging process. It is often difficult to ascertain whether redox PTMs of protein thiols have beneficial or detrimental effects on protein and cellular function, as exemplified by Prdxs, which are “gerontogenes” that can prevent the age-related decline in cognitive function and can increase healthspan and lifespan (Neumann et al., 2003; Oláhová et al., 2008; Timmermann et al., 2010; Kim et al., 2011; Nyström et al., 2012). Indeed, because Prdxs are overoxidized with age (which progressively inactivates their antioxidant activity), redox PTM of Prdxs was initially considered as a detrimental factor contributing to aging (Hall et al., 2009; Musicco et al., 2009; Molin et al., 2011; Nyström et al., 2012). However, because overoxidation of Prdxs also favors their chaperone activity, it was later suggested that redox PTM of Prdxs may constitute a positive adaption that helps counteract the detrimental protein aggregation associated with aging (Nyström et al., 2012). Somewhat related, redox-dependent dimerization of Prdx2 drives a Prdx2-dependent increase in overall life expectancy in *C. elegans* (De Haes et al., 2014), suggesting that redox PTM of Prdx2 plays a role in counteracting the aging process. Therefore, further studies are required to fully grasp the role played by redox PTMs of protein thiols in brain aging and to explore whether redox PTMs may have different effects (i.e., beneficial or detrimental) at different stages of the aging process.

In the past few decades, additional factors have been implicated in aging including low-grade chronic inflammation (“inflammaging”; Franceschi et al., 2007; von Bernhardi et al., 2015). Which one of the various factors contributing to the aging process is the main initiator of brain aging remains unclear. There are nevertheless many lines of evidence that suggest that these various contributing factors exacerbate each other, and that cellular redox dysregulation may be a common denominator (De la Fuente and Miquel, 2009; Ortuño-Sahagún et al., 2014; Yin et al., 2016). As a possible underlying molecular mechanism, it has been suggested that redox PTM of NF-kB, a pivotal transcription factor regulating inflammation, modulates NF-kB activity and subsequently the inflammatory response during aging (Ljubuncic et al., 2010; Zhang et al., 2013). Indeed, under sustained O/N stresses (as seen in aging), tyrosine residues of Iκ*B*α (one of NF-kB subunits) are nitrated, which leads to a prolonged and excessive NF-kB activation and signaling (Adler et al., 2007; Yakovlev et al., 2007). More integrated studies of the complex process of aging would be required to understand the interconnection and crosstalk between redox PTMs of protein and the various factors contributing to brain aging.

## Role of Redox PTMs of Proteins in Neurodegenerative Conditions

In age-related neurodegenerative conditions such as AD, PD, and ALS, an increase in O/N stresses and associated redox PTMs has consistently been described; S-nitrosated, S-sulfonylated, nitrated and carbonylated proteins are amongst the redox PTMs detected in the cerebrospinal fluid, plasma and postmortem brains and spinal cords of patients with AD, PD or ALS (Markesbery, 1997; Jenner, 2003; Mitsumoto et al., 2008; Riederer et al., 2009; Di Domenico et al., 2011; Sultana et al., 2011a,b; Barone et al., 2012; Sultana and Butterfield, 2013; Zahid et al., 2014). Crucially, these O/N stresses-induced PTMs are also observed at the presymptomatic stages of neurodegenerative diseases (Andrus et al., 1998; Aluise et al., 2010, 2011; Butterfield et al., 2010, 2012; Hartl et al., 2012; Granold et al., 2015; Shen et al., 2015). For example, redox proteomics on postmortem brains from patients with preclinical AD (presymptomatic stage) or with AD with mild cognitive impairment (symptomatic stage) has shown that protein carbonylation increases at the transition between the two clinical stages and correlates with clinical features, pathology and biochemistry of AD (Butterfield et al., 2010, 2012; Sultana et al., 2010; Aluise et al., 2011). Together, this suggests that O/N stresses-induced PTMs are not simply a late consequence of the disease progression but that they play an active role in the pathophysiology of neurodegenerative conditions.

Redox PTMs contribute to neurodegenerative conditions by facilitating the aggregation of disease-associated proteins. For instance, redox PTMs such as oxidation, sumoylation, and nitration of amyloid β (Aβ) or α-synuclein (associated with AD and PD, respectively) inhibit their degradation by the ubiquitin/proteasome system and autophagy, which leads to further accumulation and aggregation of their toxic products (Kuo et al., 1998; Przedborski et al., 2001; Boutte et al., 2006; Butterfield et al., 2010; Stefanis, 2012; Chavarría and Souza, 2013; Barrett and Timothy Greenamyre, 2015; Roher et al., 2017; Savyon and Engelender, 2020).

Also, aberrant redox PTMs of proteins playing key roles in cell physiology are consistently observed in neurodegenerative conditions (Nakamura et al., 2013; Akhtar et al., 2016; Di Domenico et al., 2017; Tramutola et al., 2017; Valle and Carrì, 2017; Dyer et al., 2019; Sbodio et al., 2019; Tegeder, 2019; Nakamura and Lipton, 2020; Figures 2C–E). For example, the redox-sensitive dynamin-related protein 1 (Drp1), a GTPase involved in mitochondrial fission, is activated by S-nitrosation (Drp1-SNO; Cho et al., 2009; Willems et al., 2015; Figure 2C). Drp1 is aberrantly S-nitrosated in neurodegenerative disease as high levels of Drp1-SNO and hyperactive Drp1 are detected in postmortem brains and peripheral blood lymphocytes of AD patients (Cho et al., 2009; Wang et al., 2009; Manczak and Reddy, 2012; Wang S. et al., 2012). Crucially, exposure of primary neuron cultures to Aβ leads to increase in Drp1-SNO, excessive mitochondrial fission, impaired energy metabolism, and neuronal cell loss, mimicking what is seen in AD, while the use of a non-nitrosylable Drp1 mutant prevents these detrimental effects (Cho et al., 2009; Wang et al., 2009; Trushina et al., 2012; DuBoff et al., 2013; Joshi et al., 2017; Pérez et al., 2017; Flannery and Trushina, 2019; Oliver and Reddy, 2019). Therefore, S-nitrosation of Drp1 plays a key role in the mitochondrial dynamic defects and neurodegeneration observed in AD (Wang et al., 2009; Cho et al., 2010, 2013; Nakamura and Lipton, 2020). The multifunctional protein PDI is also aberrantly S-nitrosated in neurodegenerative conditions as shown in postmortem spinal cords of ALS patients and transgenic SOD1^G93A^ mouse model of ALS as well as in postmortem brains of AD and PD patients (Uehara et al., 2006; Walker et al., 2009; Chen et al., 2013; Jeon et al., 2014; Conway and Harris, 2015; Figure 2D). PDI is an ER protein, but in disease conditions, triggered by redox PTMs in particular, it can translocate to the cytoplasm where it localizes to inclusions containing ALS-associated proteins (Turano et al., 2002; Honjo et al., 2011; Walker and Atkin, 2011; Farg et al., 2012; Jeon et al., 2014; Valle and Carrì, 2017; Matsusaki et al., 2020; Parakh et al., 2020). PDI, as an oxidoreductase chaperone, catalyzes the maturation of disulfide bond-containing proteins through oxidation and isomerization functions (Gonzalez et al., 2010). However, S-nitrosation of PDI Cys residues in its two thioredoxin-like domains inactivates both its chaperone and isomerase activities, which leads to the accumulation of misfolded proteins, in particular ALS-associated proteins like SOD1, and to a persistent ER stress that ultimately triggers neuronal injury and death (Uehara et al., 2006; Walker et al., 2009; Chen et al., 2012, 2013; Jeon et al., 2014; Medinas et al., 2018; Matsusaki et al., 2020). Similarly, excessive levels of S-nitrosation of another multifunctional protein, Prdx2, are observed in dopaminergic neurons differentiated from induced pluripotent stem cells from PD patients as well as in PQ/MB-exposed PD mouse model and postmortem brains of patients with PD (Fang et al., 2007; Sunico et al., 2016). Importantly, S-nitrosation of Prdx2 breaks the normal redox cycle that regenerates Prdx2, inhibits its peroxidase activity, and reduces its overoxidation (which is essential for stabilization of Prdx2 chaperone complexes; Jang et al., 2004; Fang et al., 2007; Engelman et al., 2013; Zhang et al., 2019). Together, this suggests that aberrant redox PTM of Prdx2 Cys residues impairs its antioxidant and chaperone functions, leading to accumulation of misfolded proteins and O/N stresses in neurodegenerative conditions. Neuroinflammation is another essential contributing factor to neurodegeneration (Hsieh and Yang, 2013; Guzman-Martinez et al., 2019). Crucially, redox PTMs of protein thiols have been implicated in inflammation regulation (Gloire and Piette, 2009; Nakamura et al., 2013; Ryan et al., 2014; Gorelenkova Miller and Mieyal, 2015). For instance, S-glutathionylation of various proteins of the NF-kB pathway can modulate the inflammatory response in different cell types, and it has been suggested that S-glutathionylation of protein thiols plays a key role in neuroinflammation in neurodegenerative conditions (Reynaert et al., 2006; Sabens Liedhegner et al., 2012; Gorelenkova Miller and Mieyal, 2015; Cha et al., 2017). Thus, redox PTMs of protein thiols can detrimentally impair the function of target proteins, which in turn can dysregulate many important pathways such as mitochondrial function, proteostasis, ER stress, neuroinflammation, and contribute to the neurodegenerative process (Sabens Liedhegner et al., 2012; Nakamura et al., 2013; Gorelenkova Miller and Mieyal, 2015; Nakato et al., 2015; Valle and Carrì, 2017; Nakamura and Lipton, 2020).

Aberrant redox PTMs have been described in several proteins that can be mutated in the familial forms of neurodegenerative conditions. Importantly, redox PTMs of non-mutated disease-associated proteins can phenocopy the effect of rare genetic mutations (Nakamura et al., 2013). For example, mutations in Parkin in patients with early-onset autosomal recessive juvenile PD impairs its E3 ubiquitin ligase activity, which leads to an abnormal accumulation of protein aggregates and cell death (Pankratz and Foroud, 2007; Nakamura et al., 2013; Arkinson and Walden, 2018; Figure 2E). Similar detrimental effects on Parkin function are observed in non-mutated Parkin modified by S-nitrosation (Chung et al., 2004; Yao et al., 2004; Sunico et al., 2013). Levels of S-nitrosated Parkin are increased in postmortem tissues of patients with sporadic PD, which implicates S-nitrosation of Parkin in the pathophysiology of these forms of PD (Chung et al., 2004; Meng et al., 2011; Sunico et al., 2013). Therefore, O/N stresses-induced PTM of protein thiols has been proposed to play an important role in the pathophysiology of the most common forms of neurodegenerative conditions (Sabens Liedhegner et al., 2012; Nakamura et al., 2013; Valle and Carrì, 2017; Dyer et al., 2019).

## The Therapeutic Potential of Redox PTMs of Proteins

Given the contribution of O/N stresses in neurodegenerative conditions, therapeutic strategies aiming at reducing O/N stresses using common free radical scavengers such as coenzyme Q10 or Vitamin E have been tested in clinical trials for these conditions, but without much success (Feng and Wang, 2012; Filograna et al., 2016). Mainly due to a lack of appropriate biomarkers, there is still little evidence however that these interventions effectively reduce O/N stresses levels in the patients’ central nervous system. Also, the antioxidants tested target specific RONS but it is not known whether the targeted RONS are implicated in neurodegenerative disease. Indeed, not all RONS are equivalent (Murphy et al., 2011) and only a subset of specific RONS is likely implicated in a given disease state. Physiological levels of RONS are essential for normal cell function and RONS can be beneficial for healthspan and lifespan (Kawagishi and Finkel, 2014; Ristow and Schmeisser, 2014; Ristow, 2014; Yun and Finkel, 2014), therefore globally reducing RONS levels may not only fail to provide therapeutic benefits but may also trigger detrimental side-effects (Kawagishi and Finkel, 2014; Nakamura and Lipton, 2016). These represent few of the factors that may have contributed to the limited success of antioxidant-based therapeutic approaches for the treatment of neurodegenerative conditions.

As an alternative redox-based therapeutic approach, it has been proposed that modulation of redox PTMs of proteins playing part in the neurodegenerative process using small molecules could help prevent disease-associated dysfunction of these proteins and restore their normal activity (Lipton et al., 2002; Wani et al., 2014; Nakamura and Lipton, 2016). This approach has been shown to provide significant therapeutic benefits in several rodent models of neurological conditions. For example, deprenyl/selegiline and its derivatives (CGP3466B, TCH346), used in early PD (Moore and Saadabadi, 2020), has been shown to bind GAPDH (Kragten et al., 1998) and prevent S-nitrosation of its catalytic site (Hara et al., 2006b). S-nitrosation is essential for GAPDH function as it increases its binding to E3 ubiquitin-protein ligase Siah1, which leads to GAPDH translocation to the nucleus and its activity as a transcriptional activator of the apoptotic cascade (Sawa et al., 1997; Kragten et al., 1998; Hara et al., 2005, 2006a,b; Sen et al., 2008). CGP3466B, by preventing GAPDH S-nitrosation, blocks the apoptotic cascade induced by S-nitrosated GAPDH. Accordingly, CGP3466B has been shown to reduce neuronal cell death in animal models of PD (Andringa and Cools, 2000; Andringa et al., 2000; Waldmeier et al., 2000; Hara et al., 2006b; Naoi et al., 2020), ALS (Sagot et al., 2000; Waldmeier et al., 2000), AD (Sen et al., 2018), muscular dystrophy (Erb et al., 2009; Yu et al., 2013), cocaine addiction (Xu et al., 2013; Harraz and Snyder, 2015), traumatic brain injury (Liang et al., 2017), and ischemia (Simon et al., 2001; Ahmari et al., 2020). Together, these studies highlight the therapeutic potential of targeting redox PTMs of specific proteins to modulate protein function and to counteract the neurodegenerative process.

Based on the fact that O/N stresses-induced PTM of proteins can affect the conformation of target proteins and their binding affinity to other proteins and potential drugs, pathologically-activated therapeutics have been developed to interact with “cryptic” sites of the target proteins that are exposed only under pathological conditions (Lipton, 2007). This approach aims to reduce the pathological activity of the target protein and to spare its normal activity in order to prevent any side-effects associated with drug treatment (Lipton, 2007). Such approach has been used to dampen the pathological hyperactivity of the N-methyl-D-aspartate receptor (NMDAR; Figure 2F). NMDAR is a glutamate-gated ion channel permeable to Ca^2+^ and is essential for neurotransmission, learning, and memory formation (Lipton, 2006). Hyperactivity of NMDAR is a common disease mechanism for many neurodegenerative conditions (Lipton and Rosenberg, 1994), and plays a critical role in excitotoxicity and synaptic damage in AD (Li et al., 2011; Talantova et al., 2013; Molokanova et al., 2014). To reduce NMDAR hyperactivity, the FDA-approved aminoadamantane compound, Memantine, an uncompetitive fast-off rate NMDAR antagonist that acts as an open-channel blocker was used initially (Chen et al., 1992; Chen and Lipton, 1997; Lipton, 2006). To improve the limited effect of Memantine on disease symptoms, and based on the observation that S-nitrosation of NMDAR reduces its activity (Lipton and Rosenberg, 1994; Choi et al., 2000; Lipton, 2006; Takahashi et al., 2007; Molokanova et al., 2014), NitroSynapsin (YQW-036, NitroMemantine), a derivative of Memantine that combines the aminoadamantane moiety and a nitro group was later synthesized and tested (Lipton, 2006, 2007; Wang et al., 2006; Talantova et al., 2013). NitroSynapsin utilizes the high-affinity Memantine binding site on NMDARs to target the nitro group for interaction with the S-nitrosation/inhibitory site of NMDAR (which is external to the Memantine-binding site); this increases NMDAR S-nitrosation and further reduces its excessive activity (Takahashi et al., 2015; Ghatak et al., 2020). Importantly, this new drug has been shown to reduce synaptic degeneration and improve the disease phenotype of a 3xTg AD mouse model (Talantova et al., 2013), a rat model of vascular dementia (Takahashi et al., 2015), a MEF2C haploinsufficiency mouse model of autism (Tu et al., 2017), a tuberous sclerosis mouse model (Okamoto et al., 2019) and an hAPP-J20 AD mouse model (Ghatak et al., 2020). Very recently, the effect of NitroSynapsin on neuronal activity has also been explored in neurons and cerebral organoids derived from human induced pluripotent stem cells (Ghatak et al., 2020). Interestingly, NitroSynapsin (but not Memantine) dampens the AD-associated increase in spontaneous action potentials and hypersynchronous network activity, suggesting that NitroSynapsin is more effective than Memantine as a human AD drug (Ghatak et al., 2020), which will be tested in future clinical trials.

The potential synergistic effect on drug efficacy of the combination of an existing drug and a nitro group has also been tested with the FDA-approved compound Clomethiazole (CMZ). CMZ is a well-established neuroprotective potentiator of the redox-sensitive gamma-aminobutyric acid receptor (GABAR; Castel and Vaudry, 2001; Wilby and Hutchinson, 2004; Gasulla et al., 2012; Dejanovic and Schwarz, 2014; Vandevrede et al., 2014; Calvo and Beltrán González, 2016). Crucially, NMZ/GT-1061, a CMZ compound analog possessing a nitro group, was shown in AD mouse models to significantly reduce Aβ deposition and cognitive decline, and to restore neuronal plasticity (Qin et al., 2012; Luo et al., 2015, 2016; Hollas et al., 2019); however, it remains unknown whether NO-mediated redox PTMs on GABAR play a part in the improved beneficial therapeutic effects provided by NMZ treatment.

Given the importance of O/N stresses and associated modifications in the onset and progression of many diseases, redox PTMs, especially irreversible redox PTMs, have been proposed as potential circulating biomarkers for a range of conditions including cardiovascular and pulmonary diseases (Di Domenico et al., 2011; Butterfield et al., 2014; Frijhoff et al., 2015; Mnatsakanyan et al., 2018; Tomin et al., 2019). Several studies have explored the proteins modified by irreversible redox PTMs (e.g., carbonylation) in cerebrospinal fluid and blood of AD and control patients and have detected differences in oxidation levels of a small number of proteins (Choi et al., 2002; Korolainen et al., 2007; Korolainen and Pirttilä, 2009; Cocciolo et al., 2012) suggesting that specific redox-modified proteins could be used as biomarkers for neurodegenerative conditions. Major technical challenges have however hindered so far the discovery of the full spectrum of proteins modified by redox PTMs in clinical samples (Delobel et al., 2016; Mnatsakanyan et al., 2018). Another major hurdle in the use of redox-modified proteins as biomarkers for neurodegenerative conditions is the limited passage of proteins or protein fragments through the blood-brain barrier (Di Domenico et al., 2011; Wang et al., 2012a; Hampel et al., 2018). So far, a handful of proteins present in biofluids of patients has been identified as potential biomarkers for neurodegenerative conditions, including Tau, Aβ, and neurofilament light (Hampel et al., 2018; Lewczuk et al., 2018; Robey and Panegyres, 2019; Ashton et al., 2020). Given that redox PTMs of these proteins contribute to the pathophysiology of neurodegenerative diseases (Horiguchi et al., 2003; Reynolds et al., 2006; Alkam et al., 2008; Kummer et al., 2011; Reyes et al., 2011), assessing the redox status of these proteins could provide crucial information, for example on the disease stage, which could potentially help follow disease progression or assess the efficacy of new drugs. It is therefore essential to further improve the sensitivity and robustness of current methodologies used to detect redox PTMs in clinical samples in order to discover redox-modified proteins that could discriminate pathological vs. healthy states and that could potentially be used as diagnostic or prognostic tools for neurodegenerative conditions.

## Conclusion

Redox PTMs of protein thiols play an important role in normal cell physiology, brain aging, and in the pathophysiology of several neurodegenerative conditions, including AD, PD, and ALS. Aberrant redox PTMs can occur in response to O/N stresses and contribute to neurodegeneration by disrupting numerous pathways, from proteostasis to mitochondrial dynamics and function. Further developments in mass spectrometry-based redox proteomics will allow to explore the full redox proteome and to identify proteins and pathways modulated by specific redox PTMs in physiology and disease. A better understanding of drug targets’ redox regulations in disease conditions would also be required to design more effective and selective redox-based therapeutic approaches for neurodegenerative conditions. Approaches that would modulate redox PTMs on specific target proteins to elicit a selective cellular response could also be tested as an alternative redox-based therapeutic strategy for neurodegenerative disease.

## Author Contributions

MF wrote and edited the entire manuscript.

## Conflict of Interest

The author declares that the research was conducted in the absence of any commercial or financial relationships that could be construed as a potential conflict of interest.
